# Phenytoin Toxicity Presenting With Ataxia and Nystagmus: A Case Report

**DOI:** 10.7759/cureus.119142

**Published:** 2026-11-04

**Authors:** Sumaya Elashmawy, Karin Kingston

**Affiliations:** 1 Geriatrics, Blackpool Teaching Hospitals NHS Foundation Trust, Blackpool, GBR

**Keywords:** acute kidney injury, ataxia, encephalopathy, geriatric medicine, nystagmus, phenobarbitone, phenytoin toxicity, primidone, therapeutic drug monitoring

## Abstract

Phenytoin has a narrow therapeutic window, and elderly patients with multiple comorbidities are more susceptible to developing toxicity. This report details a patient who developed cerebellar symptoms during admission. Elevated phenytoin levels were identified and were higher still when corrected for low serum albumin. Structural, central infective, and epileptic causes were excluded. Despite repeated dose adjustments, therapeutic levels could not be maintained. Establishing therapeutic levels may have been further complicated by declining renal function and concurrent infection, and phenobarbitone levels were also supratherapeutic. He deteriorated and subsequently died. This case highlights the need for early phenytoin level monitoring when toxicity is suspected, particularly in frail patients with hypoalbuminemia, renal impairment, or acute illness, where dose titration is challenging.

## Introduction

Phenytoin is an antiseizure drug with a narrow therapeutic index [1]. It works by modulating voltage-gated sodium channels to control neuronal firing [2], and it is effective for generalized tonic-clonic seizures, complex partial seizures, and status epilepticus without causing significant neurological impairment [3]. Prolonged use at therapeutic range can cause gingival hyperplasia, rashes, and sedation, but supratherapeutic levels can lead to cerebellar manifestations, hypotension, and cardiac arrhythmias that potentially endanger life [3]. Excess phenytoin suppresses the central nervous system, producing a cerebellar syndrome of ataxia, nystagmus, hand tremor, and diplopia, with severity depending on the dose and blood levels of the drug [1].

Older patients are particularly vulnerable to toxicity. Advanced age, renal or hepatic impairment, hypoalbuminemia, drug interactions, and acute illness can all raise free drug concentration or reduce clearance independent of dose [1]. In this group, toxicity is easily missed, because ataxia, falls, and drowsiness are often attributed to frailty, infection, or existing comorbidity rather than to the drug. Total serum levels can also be misleading when albumin is low, and levels may remain difficult to control even after the dose is reduced.

Although phenytoin toxicity presenting as ataxia has been described, there is limited evidence on how it unfolds in frail older patients who also have acute infection and renal dysfunction, in whom a stable therapeutic level may be hard to achieve. This report describes such a case, to highlight the diagnostic and management challenges and the importance of early drug-level monitoring in patients on phenytoin who present with falls.

## Case presentation

A 69-year-old man with long-standing epilepsy had been maintained on phenytoin 500 mg/day in three divided doses (200 mg in the morning, 100 mg at midday, and 200 mg in the evening) and primidone 500 mg each morning, according to the medication reconciliation on hospital admission. Total phenytoin levels of 7.2-13.1 mg/L had been recorded between 2017 and 2020, but the dose at that time and adherence before admission were not documented. His other comorbidities were orthostatic hypotension, ischemic cardiomyopathy with left ventricular dysfunction, heart failure, chronic obstructive pulmonary disease, and a permanent pacemaker. He had mild baseline cognitive impairment, lived independently, and had a Clinical Frailty Scale (CFS) score of 4 [4]. He had been admitted to a rehabilitation unit because of recurrent falls secondary to postural hypotension.

He then had an unwitnessed fall at the rehabilitation unit and was taken to the emergency department (ED). Neurological examination at that point was normal, and a computed tomography (CT) scan of his head showed no acute abnormality (Figure 1). He had minimal wheeze and bilateral fine basal crepitations and was treated for respiratory syncytial virus (RSV) pneumonitis. Because of recurrent falls, frailty, and postural hypotension, he was admitted to a geriatrics ward. Day 1 was the day of hospital admission.

**Figure 1:**
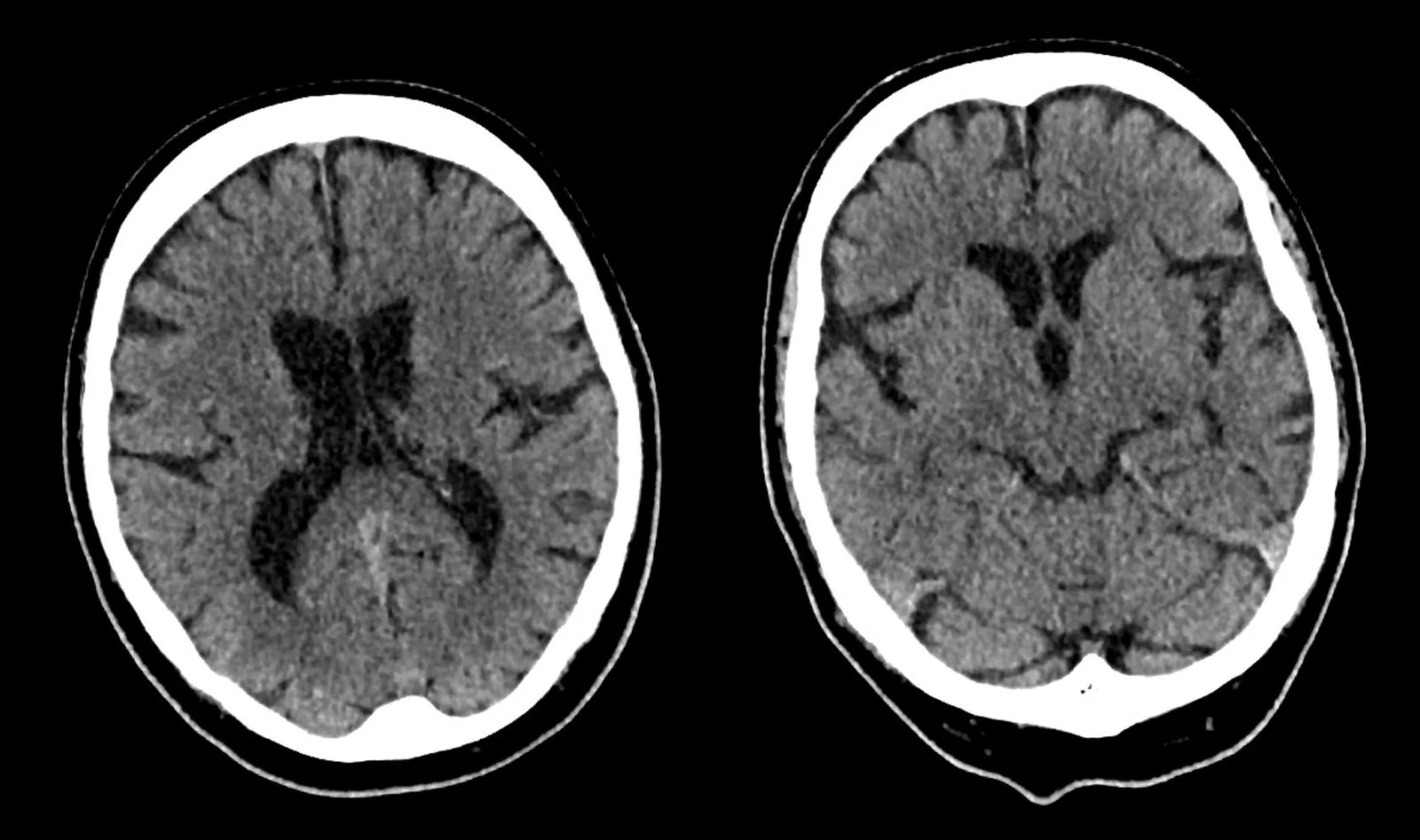
Axial CT of the head demonstrating no evidence of acute intracranial hemorrhage, mass effect, or infarct CT: computed tomography

During admission, he developed new cerebellar signs, such as ataxia, dysdiadochokinesia, bidirectional horizontal nystagmus, and past-pointing, together with marked drowsiness. Phenytoin toxicity was suspected, and the serum phenytoin level was 28.3 mg/L (reference range: 5-20 mg/L) on day 12. Neurology review on day 18 confirmed the diagnosis. By then, his level of consciousness had fallen (Glasgow Coma Scale (GCS) score 9: E2, V2, M5 [5]), so coordination and gait could not be assessed, and fundoscopy was not possible. There was no meningism, myoclonic jerks, hippus, or focal epileptic activity, and on head thrust testing, no obvious nystagmus was visualized, although nystagmus had been documented earlier in the admission. Because his drowsiness persisted despite dose adjustment and inflammatory markers were raised, the neurologist requested urgent phenytoin, primidone, and phenobarbitone levels to establish his total antiseizure medication burden, advised repeating the phenytoin level in 48 hours, and recommended a lumbar puncture to rule out central nervous system (CNS) infection or CNS pathology.

Investigations

An electroencephalogram (EEG) was done, which showed diffuse 2-5 Hz slow-wave activity consistent with moderate encephalopathy and no epileptiform discharges, excluding non-convulsive status epilepticus. Furthermore, lumbar puncture excluded CNS infection: clear cerebrospinal fluid (CSF), no pleocytosis, and sterile culture. Mildly elevated protein (1.22 g/L) was attributed to systemic inflammation. Magnetic resonance imaging (MRI) of the brain (Figure 2) showed no acute intracranial abnormality. Total (not free) serum phenytoin concentrations were measured throughout, interpreted against the laboratory reference range of 5-20 mg/L; levels were obtained as random samples rather than at a standardized time relative to dosing (see Table 1). Albumin-corrected estimates are also shown in Table 1 (formula in the Table 1 legend). Corresponding renal function (including estimated glomerular filtration rate (eGFR)), liver function, electrolytes, white cell count (WCC), C-reactive protein (CRP), and serum albumin were also trended throughout admission (see Table 2). Serum albumin was persistently and markedly reduced (24-29 g/L; reference range: 35-50 g/L) at every measured time point. Serum phenobarbitone was measured at the neurologist's request. This assay is not available locally, so samples were sent externally, and results took several days to return. All four samples were above the reference range of 10-40 mg/L: 46.4 mg/L (sample taken on day 18), 44.9 mg/L (day 21), 43.6 mg/L (day 22), and 45.3 mg/L (day 26); the last result was reported after his death.

**Figure 2:**
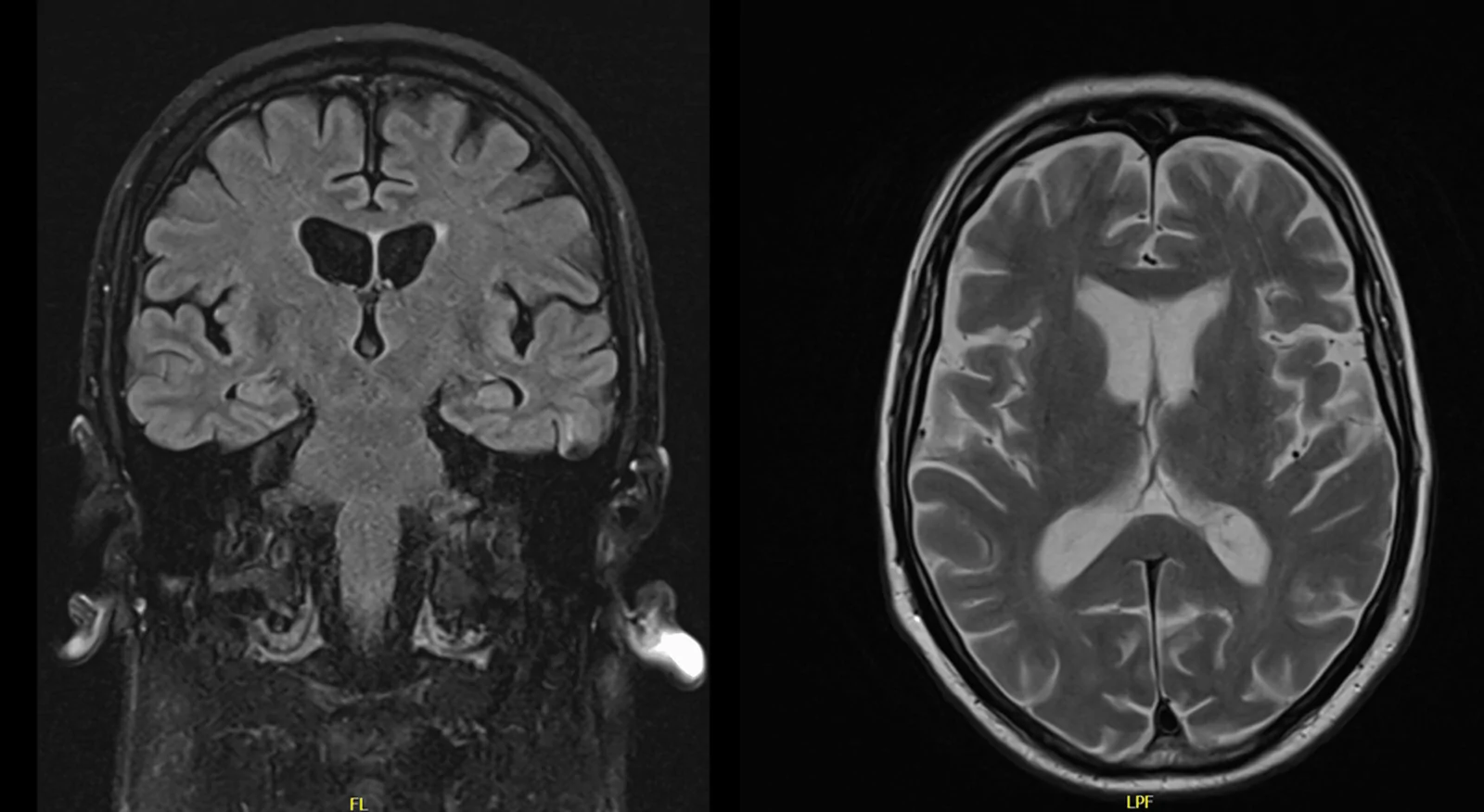
MRI of the head Coronal FLAIR (left) and axial T2-weighted (right) MRI of the brain images showing no acute intracranial abnormality, excluding a structural cause for the patient's cerebellar signs. MRI: magnetic resonance imaging; FLAIR: fluid-attenuated inversion recovery

**Table 1:** Serial total and albumin-corrected serum phenytoin levels (reference range: 5-20 mg/L) Serial total and albumin-corrected serum phenytoin levels on days after admission (day 1=day of admission). *Corrected level = measured total level / (0.2 × albumin [g/dL] + 0.1), the formula for hypoalbuminemia (see Discussion); albumin in g/L was divided by 10. †Albumin was not measured on day 17; the range uses the albumin of day 16 (29 g/L) and day 18 (25 g/L). Corrected values are estimates; free phenytoin was not measured. AKI: acute kidney injury; OD: once daily

Timeline	Total phenytoin (mg/L)	Albumin (g/L)	Albumin-corrected phenytoin (mg/L)*	Dose and clinical context
2017-2020	7.2-13.1	-	-	Historical total levels within range; dose at that time not documented
Day 12	28.3 ↑	28	42.9 ↑	Ataxia, nystagmus, past-pointing. Dose reduced from 500 mg/day (200/100/200 mg) to 450 mg OD from day 13
Day 16	24.4 ↑	29	35.9 ↑	AKI peak (creatinine 215 µmol/L); aspiration pneumonia. Dose reduced to 400 mg OD (single intravenous dose this day, oral thereafter)
Day 17	24 ↑	Not measured	35.3-40 ↑†	Drowsy and delirious
Day 20	19.3	26	31.1 ↑	Total level within range, corrected estimate supratherapeutic; clinically still drowsy
Day 22	20.5 ↑	24	35.3 ↑	Total level marginally above range
Day 26	21.9 ↑	27	34.2 ↑	Rising on unchanged 400 mg OD
Day 27	24 ↑	25	40 ↑	Persistently elevated; urgent neurology review
Day 28	19.6	25	32.7 ↑	Total level within range, corrected estimate supratherapeutic

**Table 2:** Serial renal function, liver function, electrolytes, albumin, and inflammatory markers Day numbering is as in Table 1. No biochemistry was sampled on day 17, when the 24 mg/L phenytoin level was taken, so day 18 is shown. Day 28 values are from the morning sample. ALP: alkaline phosphatase; ALT: alanine transaminase; Na: sodium; K: potassium; WCC: white cell count; CRP: C-reactive protein; eGFR: estimated glomerular filtration rate; NR: normal reference range; AKI: acute kidney injury; OD: once daily

Timeline	Creatinine (µmol/L) NR 64-104	eGFR (ml/min/1.73m²)	Urea (mmol/L) NR 2.5-7.8	ALP (IU/L) NR 30-130	ALT (IU/L) NR 0-49	Na (mmol/L) NR 133-146	K (mmol/L) NR 3.5-5.3	Albumin (g/L) NR 35-50	WCC (×10⁹/L) NR 3.6-10.5	CRP (mg/L) NR 0.2-4.9
Day 12	104	63	8.8 ↑	102	12	138	3.5	↓28	10	Not tested
Day 16	↑215	26	↑18.3	111	10	138	4	↓29	↑12.2	↑91.8
Day 18	↑138	44	↑15.8	99	10	140	3.8	↓25	9.3	↑83.9
Day 20	↑107	60	7.7	101	11	144	Hemolyzed	↓26	8.6	↑56.5
Day 22	75	88	4.3	91	15	140	3.5	↓24	↑12.1	↑28.8
Day 26	64	>90	3.2	111	12	139	↓3.1	↓27	10.5	↑24.7
Day 27	↓58	>90	3.4	94	11	136	↓3.2	↓25	↑11.5	↑32.3
Day 28	67	>90	4	95	12	140	↓3.4	↓25	↑11.8	↑47.3

Treatment and outcome

With advice and assessment from the neurology consultant, phenytoin dosage was reduced in steps from 500 mg/day (three divided doses) to 450 mg once daily on day 13 and 400 mg once daily on day 16 (Table 1). Apart from a single intravenous dose on day 16, phenytoin was given orally, and the dose was not split. However, despite multiple dose adjustments, total phenytoin levels mostly remained supratherapeutic. Worsening renal function (acute kidney injury, from day 16) and intercurrent infection (RSV pneumonitis, followed by aspiration pneumonia) may also have contributed to the difficulty establishing a therapeutic range, although the first toxic level preceded the acute kidney injury.

The patient worsened throughout admission, developing reduced consciousness and poor oral intake. He subsequently developed large bowel pseudo-obstruction. Phenytoin was continued at 400 mg once daily until comfort-focused care began in the early morning of day 29. He died later that day and was referred to the coroner for post-mortem examination; results were not available at the time of writing.

## Discussion

Phenytoin is a first-line antiepileptic medication used to manage various epilepsy syndromes [6]. The precise mechanism of toxicity remains unclear, but one theory suggests it may result from phenytoin-induced neuronal apoptosis, potentially due to increased reactive oxygen species production, leading to oxidative stress, mitochondrial dysfunction, and potential interference with neural plasticity [2]. Some studies also report cerebellar ataxia as a chronic adverse effect even at therapeutic range [6].

The neurotoxic effects of phenytoin are concentration-dependent, although the reported thresholds are derived from mean values across populations of patients with epilepsy and represent population-level estimates rather than individualized cut-offs [7]. Occasional mild nystagmus is estimated to present at levels between 10 and 20 mg/L and ataxia at levels of 30-40 mg/L [7]. Higher concentrations of 40-50 mg/L are associated with lethargy, confusion, and hyperactivity, and levels exceeding 50 mg/L carry a risk of coma and seizures [7]. Total phenytoin levels in this patient did not exceed 30 mg/L (Table 1), yet he developed ataxia and progressive drowsiness. As a single case, this observation cannot be generalized to redefine toxicity thresholds; rather, it illustrates that published reference ranges are averages drawn from diseased cohorts and considerable inter-individual variability exists around them. This reinforces the importance of clinical correlation alongside serum levels, rather than reliance on concentration thresholds in isolation, when assessing for toxicity.

A further possible contributing factor in this case is the patient's persistently low serum albumin, which remained between 24 and 29 g/L throughout the admission, well below the reference range of 35-50 g/L. As approximately 90% of circulating phenytoin is protein-bound, predominantly to albumin, and only the unbound fraction is pharmacologically active, hypoalbuminemia increases the free phenytoin fraction independent of the total serum concentration routinely measured. This means the total phenytoin levels reported in this case may have underestimated the true degree of active drug exposure, and the corrected phenytoin level formula (accounting for serum albumin) may be more clinically representative than the uncorrected total level in patients with hypoalbuminemia [7]. Using the formula for hypoalbuminemia, in which the corrected level equals the measured level divided by (0.2 × albumin [g/dL] + 0.1) [7], albumin-corrected estimates reached 42.9 mg/L on day 12 and 40 mg/L on day 27 and exceeded 30 mg/L at every time point, including days 20 and 28 when the total level was within the reference range (Table 1). These estimates lie in the ranges associated with ataxia (30-40 mg/L) and with lethargy and confusion (40-50 mg/L) [7], although those ranges are defined for total levels, so the comparison is approximate. They are estimates only, as free phenytoin was not measured. This is a plausible additional mechanism contributing to the patient's clinical toxicity despite serum phenytoin concentrations that, at points, appeared only marginally elevated or within range.

Liver function remained normal throughout admission, with alanine transaminase (ALT) and alkaline phosphatase (ALP) within reference range at every measured time point (Table 2), which argues against hepatic impairment as a cause of reduced phenytoin clearance, given that phenytoin is predominantly metabolized hepatically [7]. However, ALT and ALP do not measure metabolic capacity and cannot exclude reduced hepatic clearance.

Total phenytoin levels rose from 19.3 mg/L on day 20 to 24 mg/L on day 27 on an unchanged oral dose of 400 mg once daily (a single intravenous dose was given on day 16). Phenytoin elimination is saturable, so its half-life lengthens as concentrations rise [7], and levels may take considerably longer than usual to settle after a dose change. The day 20 level may therefore have reflected earlier, higher exposure, and the later rise may reflect the patient approaching a steady state at 400 mg that was itself supratherapeutic. Random sampling times may also have contributed. The fall to 19.6 mg/L on day 28 on the same dose is not explained by these hypotheses, and none of these explanations can be confirmed from the available data.

While there are no specific guidelines for phenytoin toxicity, it is recommended that serial phenytoin levels be repeated and trended [7]. Neuroimaging in the form of CT or MRI is also often performed. Studies report CT and MRI scans showing cerebellar dysfunction and cerebellar atrophy [6]. This is contradictory to the neuroimaging findings of the patient discussed in the case report, as both CT and MRI scans were normal.

Management of phenytoin toxicity is dependent on the clinical context. A case report by Robertson et al. describes a presentation similar to the case discussed above. The authors of this case explain that the patient had complete resolution of symptoms on dose reduction [8]. Similarly, a different case report of a patient with a phenytoin level of 30 mg/L also describes the established complete resolution of symptoms within five days of dose tapering and complete cessation of medication [9].

We reviewed the patient's medication chart for interactions with phenytoin listed in the British National Formulary (BNF) [10] (Table 3). Primidone was the only drug whose effect on phenytoin concentration was not stated as a decrease: the BNF describes a bidirectional interaction and advises monitoring both drugs. Two listed interactions would be expected to lower phenytoin levels, namely, enteral feeding via a nasogastric (NG) tube, which reduces absorption, and folic acid, contained in Forceval; both were started on day 21. Total levels nevertheless rose between days 20 and 27, so these interactions do not explain the rise. The remaining interactions listed act in the opposite direction, with phenytoin reducing exposure to the other drug, and would not explain toxicity. Omeprazole, aspirin, candesartan, magnesium, and phosphate had no listed interaction. Other medications on the chart (bisoprolol, spironolactone, dapagliflozin, enoxaparin, inhaled and nebulized bronchodilators, carbocisteine, laxatives, senna, thiamine, potassium supplements, and the antibiotics piperacillin-tazobactam and co-amoxiclav) were not individually checked, so a contribution from these cannot be excluded.

**Table 3:** Medications reviewed for interactions with phenytoin (BNF) Interactions as listed in the BNF [10]; severity is the BNF's grading, and all listed interactions are graded as study evidence. Folic acid is the BNF entry for the folic acid content of Forceval. Other medications on the chart were not individually checked. BNF: British National Formulary; NG: nasogastric

Medication	Dose and days given	Interaction with phenytoin listed in the BNF (severity)	Relevance to phenytoin levels
Primidone	500 mg each morning, long-term; 450 mg from day 26	Phenytoin increases primidone concentration, and primidone affects phenytoin concentration; monitor both (moderate)	Possible contributor (see text on phenobarbitone)
Forceval (contains folic acid)	1 tablet daily from day 21	No entry for Forceval; folic acid is predicted to decrease phenytoin concentration; monitor concentration and adjust dose (severe)	Would be expected to lower levels
Enteral feed (Jevity Plus HP via NG tube)	Days 21-29	Enteral feeds decrease phenytoin absorption; manufacturer makes no recommendation (severe)	Would be expected to lower levels
Prednisolone	30 mg daily, days 4-8	Phenytoin is predicted to decrease exposure to prednisolone; monitor and adjust dose (moderate)	Effect on prednisolone, not a cause of phenytoin toxicity
Doxycycline	100 mg twice daily, days 2-7	Phenytoin decreases doxycycline concentration; adjust dose (moderate)	Effect on doxycycline, not a cause of phenytoin toxicity
Furosemide	40 mg, from admission	Phenytoin decreases the effects of furosemide; no recommendation (moderate)	Effect on furosemide, not a cause of phenytoin toxicity
Paracetamol	As required, from admission	Phenytoin decreases exposure to paracetamol; no recommendation (moderate)	Effect on paracetamol, not a cause of phenytoin toxicity
Omeprazole, aspirin, candesartan, magnesium, phosphate	Various	No interaction found	None

Serum phenobarbitone was supratherapeutic on all four samples (46.4, 44.9, 43.6, and 45.3 mg/L; reference range: 10-40 mg/L) and may have contributed to drowsiness independently of phenytoin. Cerebellar ataxia in chronic multi-drug antiepileptic therapy has been associated with higher serum phenobarbital levels [6]. Because the assay is not available locally, results took several days to return, and the last result (day 26 sample) was reported after his death. Primidone was reduced from 500 mg to 450 mg daily on day 26 on the advice of the regional neurology team because of persistently high drug levels.

Though there is literature on phenytoin toxicity presenting as ataxia, there is limited evidence of the impact of deteriorating renal function and concurrent infection, which may have affected the presentation of the patient. In this case, the first toxic level (day 12) preceded the acute kidney injury (day 16), so renal dysfunction cannot explain the initial toxicity. Because phenytoin is cleared mainly by hepatic metabolism, with only 1-5% excreted unchanged in the urine [7], renal impairment is unlikely to have reduced its clearance directly. Renal dysfunction and infection (CRP 91.8 mg/L on day 16, Table 2) are therefore considered possible contributors only, perhaps through altered protein binding, as uremia and hypoalbuminemia increase the unbound fraction [7]; a causal role cannot be established. This shows the importance of early recognition and management of phenytoin toxicity. It emphasizes the relevance of considering toxicity in patients on phenytoin presenting with frequent falls.

Causality was assessed using the Naranjo Adverse Drug Reaction Probability Scale [11] (Table 4). The score was 4, indicating a possible reaction. The score was limited by the absence of a dechallenge response, since phenytoin was reduced but never stopped, and by competing explanations for his encephalopathy, including infection, acute kidney injury, and supratherapeutic phenobarbitone, rather than by doubt over the elevated drug levels.

**Table 4:** Naranjo Adverse Drug Reaction Probability Scale applied to phenytoin Scoring as described in the Naranjo scale [11]: 9 or more, definite; 5-8, probable; 1-4, possible; and 0 or less, doubtful.

Question	Answer	Score	Basis
1. Previous conclusive reports of this reaction?	Yes	+1	Well documented [1,7-9]
2. Did the event appear after the drug was given?	Yes	+2	Cerebellar signs developed during phenytoin therapy
3. Improved when the drug was stopped or an antagonist given?	Do not know	0	Phenytoin was reduced but not stopped; no clinical improvement documented
4. Reappeared on readministration?	Do not know	0	No rechallenge
5. Alternative causes that could on their own have caused the reaction?	Yes	−1	Infection, acute kidney injury, supratherapeutic phenobarbitone
6. Reappeared with placebo?	Do not know	0	Not applicable
7. Drug detected in toxic concentrations?	Yes	+1	Total levels up to 28.3 mg/L; corrected estimates higher (Table 1)
8. More severe with higher dose or less severe with lower dose?	No	0	No clear change in levels or clinical state with dose reduction
9. Similar reaction on previous exposure?	Do not know	0	No previous toxicity documented
10. Confirmed by objective evidence?	Yes	+1	Supratherapeutic levels; nystagmus on examination
Total	-	4	Possible (1-4)

Limitations of this case include the absence of serial GCS scores and detailed baseline functional assessment; no documentation of adherence or of dosing before admission, including the dose when the 2017-2020 levels were taken; free phenytoin levels not measured; phenobarbitone results delayed by external testing, the last reported after death; no systematic interaction review of all medications; and post-mortem results not available at the time of writing.

## Conclusions

This case demonstrates the importance of checking serum phenytoin levels in patients with suspicion of phenytoin toxicity. Hypoalbuminemia meant that total levels may have underestimated active drug exposure. Renal dysfunction, infection, and supratherapeutic phenobarbitone are possible contributors to his deterioration and to the difficulty in establishing a stable phenytoin level, although a causal relationship cannot be established from a single case (Naranjo score 4, possible). Prompt assessment of prior falls and early phenytoin levels enables appropriate titration and facilitates management.
